# Multi-drug resistance 1 genetic polymorphism and prediction of chemotherapy response in Hodgkin's Lymphoma

**DOI:** 10.1186/1756-9966-30-68

**Published:** 2011-07-16

**Authors:** Nizar M Mhaidat, Osama Y Alshogran, Omar F Khabour, Karem H Alzoubi, Ismail I Matalka, William J Haddadin, Ibraheem O Mahasneh, Ahmad N Aldaher

**Affiliations:** 1 Clinical Pharmacy Department, Faculty of Pharmacy, Jordan University of Science and Technology, Irbid, 22110, Jordan; 2 Molecular Genetics, Faculty of Applied Medical Sciences, Jordan University of Science and Technology, Irbid, 22110, Jordan; 3 Pathology Department, Faculty of Medicine, Jordan University of Science and Technology, Irbid, 22110, Jordan; 4 Histology and Cytology Department, Princess Iman Center for Research and Laboratory Sciences, King Hussein Medical Center, Amman, 11855, Jordan; 5 Hematology and Oncology Department, Jordanian Royal Medical Services, 11855, Amman, Jordan

**Keywords:** Lymphoma, C3435T SNP, MDR-1

## Abstract

**Background:**

The human multi-drug resistance gene (*MDR1*), which encodes the major trans-membrane transporter P-glycoprotein (P-gp), was found to be associated with susceptibility to cancer and response to chemotherapy. The C3435T Polymorphism of *MDR1 *gene was correlated with expression levels and functions of P-gp. Here, we studied the association between *MDR1 *C3435T polymorphism and susceptibility to Hodgkin lymphoma (HL) and patient's response to ABVD chemotherapy regimen.

**Methods:**

a total of 130 paraffin embedded tissue samples collected from HL patients were analyzed to identify the C3435T polymorphism. As a control group, 120 healthy subjects were enrolled in the study. The C3435T Polymorphism was genotyped by polymerase chain reaction and restriction fragment length polymorphism (PCR-RFLP) method. Data analysis was carried out using the statistical package SPSS version 17 to compute all descriptive statistics. Chi-square and Fisher exact tests were used to evaluate the genotype distribution and allele frequencies of the studied polymorphism.

**Results:**

these studies revealed that the frequency of T allele was significantly higher in HL patients compared to the controls (P < 0.05). In addition, the frequency of CT and TT genotypes were also significantly higher in HL patients compared to the controls (P < 0.05). No association between C3435T polymorphism and response to ABVD was detected among HL patients (P > 0.05).

**Conclusions:**

these results suggest that *MDR1 *C3435T polymorphism might play a role in HL occurrence; however this polymorphism is not correlated with the clinical response to ABVD.

## Background

Lymphomas are heterogeneous group of hematological malignancies that arise from malignant transformation of immune cells and account for 17% of all cancers in teenagers, and around 10% of childhood cancers [1]. Lymphomas are classified into two main types, Hodgkin's lymphoma (HL) and non-Hodgkin's lymphoma (NHL). The incidence of HL has risen gradually over the last few decades, representing a bimodal incidence peak, in early and late adulthood [1].

Several modalities are available to improve the overall survival in HL patients including radiotherapy, chemotherapy or combination of both [2]. However, the most commonly used regimen in the treatment of advanced stages of HL is the ABVD regimen containing doxorubicin (adriamycin), bleomycin, vinblastine and darcarbazine [3]. While more than 70% of HL patients are cured after treatment [3], about 30% of them might experience relapse after achieving initial complete remission (CR) [4]. This was attributed to the development of drug resistance, which might result from change in drug target sites or increased drug efflux by overexpression of drug transporters [5-7].

The multi-drug resistance (MDR) protein is a transporter that plays a primary role in drug resistance by affecting drug transport to cancer cells. MDR1 protein, called P-glycoprotein (P-gp), belongs to ATP-binding cassette superfamily [8]. A number of polymorphisms in the *MDR1 *gene were found to be of clinical importance, since they can alter drug absorption, distribution and elimination [9]. For example, the *MDR1 *C3435T polymorphism has been shown to affect the efficiency of chemotherapy in patients with lymphoproliferative diseases in a sample of the Europeoids of west Serbia [10].

While the association between the *MDR1 *C3435T polymorphism and NHL is well documented, the association between this polymorphism and HL has not been examined yet. In the present study, we investigated the association between the *MDR1 *C3435T polymorphism and the risk to develop HL, as well as the clinical response to ABVD chemotherapy regimen.

## Methods

### Studied groups

A total of 130 samples of paraffin-embedded tissue collected from HL patients were obtained from the Departments of Pathology at both Royal Medical Services and King Abdullah University Hospital. Patients included in the study are those of age more than 15-year old with HL, who received only ABVD regimen as initial chemotherapy. Patients were divided into two groups; complete response (n = 96) and relapsed disease (n = 34) according to International Workshop Criteria (IWC) [11].

Complete response (CR) was defined as 1) complete disappearance of all detectable evidence of disease on computed tomography (CT), 2) all disease-related symptoms, 3) normalization of biochemical abnormalities, 4) normal bone marrow biopsy, and 5) regression of nodes on CT of more than 1.5 cm in their axial diameter to less than 1.5 cm, and nodes of 1.1-1.5 to less than 1 cm. Relapsed disease (RD) was defined as: 1) the appearance of any new lesion 2) or increase in the size of more than 50% of previously involved sites or nodes in patients who achieved CR or Cru (uncertain). CRu corresponds to CR criteria but with a residual mass more than 1.5 cm in greatest axial diameter that has regressed by more than 75% [11].

Peripheral blood samples were collected from 120 healthy young volunteers as a control group from the same patient's geographical areas. Informed written consents were obtained from the participants in accordance with the requirements of the Institutional Review Boards of Jordan University of Science and Technology.

### DNA extraction

DNA was extracted from paraffin embedded tissue samples using QIAamp DNA FFPE Tissue Kit (QIAGEN, California, USA) according to standard protocol provided by the manufacturer. Approximately, 3-5 sections of 5 μm thick were cut from each sample and used for DNA extraction. Venous blood samples were collected in EDTA tubes and obtained from young healthy control group. DNA was extracted from all blood samples using Promega wizard genomic DNA purification kit (Promega, Madison, USA). DNA samples were stored at -20°C until used.

### Genotyping

The polymorphism C3435T was analyzed using polymerase chain reaction and restriction fragment length polymorphism (PCR-RFLP) method. Desired DNA target sequence (197) was amplified as described by Cascorbi *et al*. [12] using a forward primer (5'-TGT TTT CAG CTG CTT GAT GG -3') and a reverse primer (5'-AAG GCA TGT ATG TTG GCC TC-3'). The reaction mixture of 25 μL contained 50 ng of genomic DNA, 0.5 μL of each primer, 12.5 μL of the green master mix, and 1.5-9.5 μL of deionized water. The reaction mixture was initially denatured at 94°C for 2 minutes, followed by 35 cycles of denaturation at 94°C for 30 s, annealing at 60°C for 30 s and extension at 72°C for 30 s. The termination elongation was performed at 72°C for 7 minutes. Successful amplification was confirmed by detection of a 197 bp band on a 2% agarose gel using a 100 bp DNA ladder. 10 μL of each PCR product was digested with 5 units of Sau3AI at 37°C overnight. The digested products were separated using 2.5% agarose gel and detected by ethidium bromide staining. Fragments obtained were 158 bp and 39 bp to the wild type genotype C/C, 197 bp to the mutant genotype T/T and 197 bp, 158 bp and 39 bp to the C/T genotype.

### Statistical analysis

Data analysis was carried out using the statistical package SPSS version 17 to compute all descriptive statistics. Chi-square and Fisher exact tests were used to evaluate the genotype distribution and allele frequencies of the studied polymorphism. A P value of < 0.05 was considered statistically significant. Hardy-Weinberg equilibrium was assessed using the chi-square test. The C3435T genotypes were found to be in Hardy- Weinberg equilibrium.

## Results

A hundred and thirty patients diagnosed with HL, the median age is 30 years, were included in the study. Fifty five percent are males and 47.7% have early stages of HL and complaining of B-symptoms. Most of the patients (76.2%) received 6 cycles of ABVD regimen. Other baseline characteristics of the patients are shown in Table 1. As a control, 120 healthy volunteers from the same geographical areas were enrolled (54% are males with median age of 23.5 years).

**Table 1:** Demographic criteria of the patients

Variable	Patients with Complete Remission (CR) N (%)	Patients with Relapsed Disease (RD) N (%)
**Number **	96	34
**Age at diagnosis**		
Median	31	27.5
15-20	16 (16.7)	17 (50)
21-30	32 (33.3)	5 (14.7)
31-40	18 (18.8)	5 (14.7)
> 40	30 (31.2)	8 (20.6)
**Gender**		
Males	50 (52.1)	21 (61.8)
Females	46 (47.9)	13 (38.2)
**Stage**		
Early stages (I &II)	41 (42.7)	20 (58.8)
Advanced stages (III & IV)	38 (39.6)	12 (35.3)
Missed data	17 (17.7)	2 (5.9)
**Presence of B symptoms**		
Yes	54 (56.3)	19 (55.9)
No	31 (32.3)	13 (38.2)
Missed data	11 (11.4)	2 (5.9)
**Bone marrow involvement**		
Yes	5 (5.2)	4 (11.8)
No	91 (94.8)	30 (88.2)
**Histology**		
Nodular sclerosis	46 (47.9)	16 (47.1)
Mixed cellularity	25 (26)	6 (17.6)
Lymphocyte rich	5 (5.2)	3 (8.8)
Lymphocyte depleted	4 (4.2)	0 (0)
Nodular lymphocyte predominance	1 (1)	5 (14.7)
Classical	7 (7.3)	4 (11.8)
Missed data	8 (8.3)	-
**Chemotherapy regimen**	ABVD: All the patients	ABVD: Initially all the patients at relapse: ICE^a ^(8), ESHAP^b ^(8), COPP^c ^(3), ABVD^d ^(8), Others: (7).
**Number of ABVD cycles**		
< 6 cycles	10 (10.4)	6 (17.6)
6 cycles	77 (80.2)	22 (64.7)
> 6 cycles	9 (9.4)	5 (14.7)

^a^Adriamycin, Bleomycin, Vinblastine, Decarbazine; ^b^Ifosfamide, Carboplatin, Etoposide; ^c^Etoposide, Cisplatin, Cytarabine, Methylprednisolone; ^d^Cyclophosphamide, Vincristine, Prednisolone, Procarbazine.

**Figure 1:**
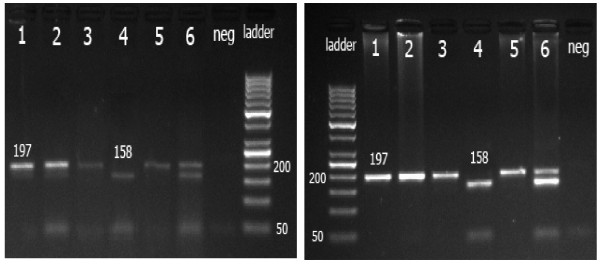
**Gel electrophoresis of C3435T polymorphism from tissue samples**. Left: The last lane from the right is 50 bp DNA ladder. Samples in lanes 1, 3 and 5 represent the PCR products and samples in lanes 2, 4 and 6, are the digest products of each sample, respectively. Sample in lane 2 is the mutant homozygous uncut TT genotype (197 bp). Sample in lane 4 represents the wild type cut CC genotype (158 bp and 39 bp). Sample in lane 6 represents heterozygous CT genotype (197 bp, 158 bp and 39 bp). Right: Gel electrophoresis of C3435T polymorphism from blood samples. The first lane from the left is 50 bp DNA ladder. Samples in lanes 1, 3 and 5 represent the PCR products and samples in lanes 2, 4 and 6, are the digest products of each sample, respectively. Sample in lane 2 is the mutant homozygous uncut TT genotype (197 bp). Sample in lane 4 represents the wild type cut CC genotype (158 bp and 39 bp). Sample in lane 6 represents heterozygous CT genotype (197 bp, 158 bp and 39 bp).

As shown in Figure 1, samples from paraffin embedded tissues and blood, were successfully genotyped using PCR-RFLP method. The mutant T allele does not carry the restriction site for *Sau3*AI enzyme and remains as 197 bp fragment, while the wild C allele cuts into two fragments of 158 and 39 bp.

Results in Table 2 revealed that both C and T alleles are common in the studied population with approximately equal distribution. However, the patient group showed significantly (*P *value < 0.05) higher frequencies of both mutant T allele (65%) and TT homozygous mutant genotype (41%) compared to the control group. This indicates that the T allele in the C3435T polymorphism is associated with and HL occurrence.

**Table 2:** Genotype and allele frequencies of C3435T polymorphism among HL patients and controls

Genotypes & Alleles	HL patients (130) N (%)	Controls (120) N (%)	P-value
CC	15 (11.5)	37 (30.8)	
CT	62 (47.7)	48 (40.0)	0.001
TT	53 (40.8)	35 (29.2)	
Allele C	92 (35.4)	122 (50.8)	0.000
Allele T	168 (64.6)	118 (49.2)	

No significant association between the C3435T genotypes (CC, CT and TT) and alleles (C and T) with patient's baseline characteristics including patient's age, gender, specimen histology, stage of the disease and presence or absence of B-symptoms (Table 3 and 4), *P *value > 0.05.

**Table 3:** Characteristics of patients according to C3435T genotypes

Characteristics	CC genotype N (%)	CT genotype N (%)	TT genotype N (%)	P-value
**Age at diagnosis**				
< 30 (n = 62)	7 (46.7)	28 (45.2)	27 (50.9)	0.823
≥ 30 (n = 68)	8 (53.3)	34 (54.8)	26 (49.1)	
**Gender**				
Males (n = 71)	7 (46.7)	29 (46.8)	35 (66)	0.095
Females (n = 59)	8 (53.3)	33 (53.2)	18 (44)	
**Histology**				
NS^a ^(n = 62)	9 (64.3)	32 (72.7)	21 (60)	0.481
MC^b ^(n = 31)	5 (35.7)	12 (27.3)	14 (40)	
**Stage**				
Early stages (I &II) (n = 61)	7 (50)	30 (58)	24 (53.3)	0.842
Advanced stages (III & IV) (n = 50)	7 (50)	22 (42)	21 (46.7)	
**Presence of B-symptoms**				
Yes (n = 73)	9 (60)	36 (64.3)	28 (60.9)	0.920
No (n = 44)	6 (40)	20 (35.7)	18 (39.1)	

^a^Nodular sclerosis; ^b^Mixed cellularity.

**Table 4:** Characteristics of patients according to C3435T alleles

Characteristics	C allele N (%)	T allele N (%)	Total	P-value
**Age at diagnosis**				
< 30	42 (45.7)	82 (48.8)	124	0.626
≥ 30	50 (54.3)	86 (51.2)	136	
**Gender**				
Males	43 (46.7)	99 (58.9)	142	0.059
Females	49 (53.3)	69 (41.1)	118	
**Histology**				
NS^a^	50 (69.4)	74 (64.9)	124	0.134
MC^b^	22 (30.6)	40 (35.1)	62	
**Stage**				
Early stages (I &II)	44 (55)	78 (54.9)	122	0.992
Advanced stages (III & IV)	36 (45)	64 (45.1)	100	
**Presence of B-symptoms**				
Yes	54 (62.8)	92 (62.2)	146	0.924
No	32 (37.2)	56 (37.8)	88	

^a^Nodular sclerosis; ^b^Mixed cellularity.

To verify whether different baseline characteristics of the patients might contribute to chemotherapy response, complete remission and disease relapse were studied according to the following criteria: age, gender, specimen histology, disease stage and presence or absence of B-symptoms (Table 5). None of these factors were associated with clinical response in HL patients (*P *value > 0.05).

**Table 5:** The correlation between clinical outcome and patient's characteristics

Baseline Factors	Complete Remission N (%)	Relapsed Disease N (%)	Total	P-value
**Age at diagnosis**				
< 30	43 (44.8)	19 (55.9)	62	0.266
≥ 30	53 (55.2)	15 (44.1)	68	
**Gender**				
Males	50 (52.1)	21 (61.8)	71	0.330
Females	46 (47.9)	13 (38.2)	59	
**Histology**				
NS^a^	46 (64.8)	16 (72.7)	62	0.490
MC^b^	25 (35.2)	6 (27.3)	31	
**Stage**				
Early stages (I &II)	41 (51.9)	20 (62.5)	61	0.309
Advanced stages (III & IV)	38 (48.1)	12 (37.5)	50	
**Presence of B-symptoms**				
Yes	54 (63.5)	19 (59.4)	73	0.679
No	31 (36.5)	13 (40.6)	44	

^a^Nodular sclerosis; ^b^Mixed cellularity.

Table 6 shows the genotype and allele frequencies of the C3435T polymorphism in HL patients with complete remission compared to those with relapse. No significant difference of CT and TT genotype distribution and allele frequency was found between the two groups (*P *value > 0.05).

**Table 6:** Genotype and allele frequencies of C3435T polymorphism among patients according to the response

Genotypes and Alleles	Complete Remission N (%)	Relapsed Disease N (%)	P-value
CC	12 (12.5)	3 (8.8)	
CT	44 (45.8)	18 (52.9)	0.729^a^
TT	40 (41.7)	13 (38.2)	
Allele C	68 (35.4)	24 (35.3)	0.986
Allele T	124 (64.6)	44 (64.7)	

^a^P value based on fisher exact test.

To identify possible correlation between the genotype and allele frequencies of the C3435T polymorphism and the progression free survival in relapsed group; patients were divided into two groups. The first include those having the relapse after one year of complete remission and the other group having the relapse during the first year of complete remission (Table 7). However, no significant difference in the frequencies of C3435T genotypes and the alleles was found. Thus, C3435T polymorphism seems to play no role in the progression free survival in the relapsed HL patients.

**Table 7:** Genotype and allele frequencies of C3435T polymorphism among the relapsed group according to progression free survival

Genotypes and Alleles	Progression free survival ≤ 1 year N (%)	Progression free survival > 1 year N (%)	P-value
CC	0 (0)	3 (18.8)	
CT	12 (66.7)	6 (37.5)	0.083^a^
TT	6 (33.3)	7 (43.7)	
Allele C	12 (33.3)	12 (37.5)	0.720
Allele T	24 (66.7)	20 (62.5)	

^a^P value based on fisher exact test.

## Discussion

In this study, we investigated for the first time whether functional polymorphism C3425T in *MDR1 *gene could affect patient's susceptibility to HL and/or modify its response to chemotherapeutic agents. The results suggest that C3435T polymorphism plays a role in susceptibility to HL but not its response to ABVD chemotherapy. We analyzed MDR1 C3435T polymorphism in DNA isolated from paraffin embedded tissues taken from patient's lymph nodes while the same polymorphism was analyzed in the controls from peripheral blood tissues. This might raise some concern that the DNA from the two tissues is not equivalent because mutations are common during cancer progression. However, unlike most other malignant tumors, HL is characterized by low number of malignant cells that are surrounded by many non-neoplastic lymphocytes (reviewed in [13]).

The results indicate approximately equal distribution of the C and T alleles of C3425T polymorphism in the Jordanian population. This distribution is similar to that of Japanese [14], Caucasian [12], Chinese [15], Polish [16] and Malay [17] populations. However, the frequency of the T allele found in the present study is higher than that reported in Taiwanese [18], African [19], Jewish [20], Iranian [21], and Polish [22] populations, but lower than that of Czech [23] and Indian [17] populations (Table 8). Thus, the distribution of C3435T polymorphism seems to fall somewhere in the middle when compared with the Asian and European populations, which might be explained by the unique geographical location of Jordan at the crossing of Asia and Europe.

**Table 8:** The frequency of 3435T allele among ethnic groups

Ethnicity	3435T allele Frequency (%)	Reference
Taiwanese (n = 110)	37.3	(Huang *et al*., 2005)
Japanese (n = 100)	49.0	(Tanabe *et al*., 2001)
Caucasians (n = 461)	53.9	(Cascorbi *et al*., 2001)
Africans (n = 206)	17.0	(Ameyaw *et al*., 2001)
Chinese in Singapore (n = 98)	54.0	(Balram *et al*., 2003)
Chinese in Mainland (n = 132)	46.6	(Ameyaw *et al*., 2001)
French (n = 227)	46.0	(Jeannesson *et al*., 2007)
Ashkenazi Jewish (n = 100)	35.0	(Ostrovsky *et al*., 2004)
Czech (n = 189)	56.5	(Pechandova *et al*., 2006)
Polish (n = 204)	52.5	(Kurzawski *et al*., 2006)
West Siberian Europeans (n = 59)	59.0	(Goreva *et al*., 2003)
Iranian (n = 300)	33.5	(Farnood *et al*., 2007)
Polish (175)	40.0	(Jamroziak *et al*., 2004)
Indians (n = 87)	63.2	(Chowbay *et al*., 2003)
Chinese (n = 96)	53.1	(Chowbay *et al*., 2003)
Malays (n = 92)	51.1	(Chowbay *et al*., 2003)
Jordanian (n = 120)	49.2	Present study

Several genetic and environmental factors such as exposure to pesticides, wood dusts and chemicals were found to be associated with development of HL [24]. In here, we observed that C3435T polymorphism is significantly associated with susceptibility to HL. The homozygous mutant TT genotype and allele T frequencies were found to be higher in HL patients. Thus, our data may indicate that the C allele of C3435T polymorphism has protective role against HL. This could be explained by the low expression of T allele compared to C allele; thereby individuals with T allele are more prone to environmental toxins and carcinogens associated with HL. Previous studies suggest that the C3435T polymorphism is in linkage disequilibrium with other *MDR1 *polymorphisms such as C1236T and G2677T in exons 12 and 21, respectively. Thus, the contribution of those polymorphisms to susceptibility to HL observed in our study cannot be ruled out. In agreement with our results, Turgut, *et al*. [25] found a significant association between C3435T polymorphism and breast cancer. In the patient group, T allele frequency was significantly higher than controls. Similarly, the TT genotype of C3435T polymorphism was found to be associated with colon cancer risk [16]. The TT genotype was also associated with other malignancies such as acute lymphoblastic leukemia [22], renal cell carcinoma [26], and other diseases as ulcerative colitis [21]. In contrast, C3435T polymorphism was not associated with breast cancer in Iranian population [27]. Furthermore, C3435T variant was also not associated with acute leukemia in Turkish patients [28] and in childhood leukemia [29]. Thus, association between C3435T polymorphism and cancer development might have a population specific component. Moreover, a study by Humeny et al. [30] showed that MDR1 C3435T polymorphism is stable during carcinogenesis. Thus, it is unlikely that the observed strong association between HL and MDR1 C3435T polymorphism is due to mutations at the examined locus that are related to cancer progression.

A variety of mechanisms that may account for resistance of cancer cells to chemotherapy were described [31]. The most important one is the increase efflux of chemotherapeutic agents outside the cells by increasing the expression level of the major membrane transporter P-glycoprotein [6]. The *MDR1 *C3435T variant was found to alter P-gp function and expression, which might affect the disease response by modifying the pharmacokinetics of anticancer drugs. Therefore, several studies have shown the effect of C3435T *MDR1 *variant on disease outcome. In our study, we investigated the effect of C3435T variant on HL outcome in patients who received ABVD regimen containing common P-gp substrates adriamycin and vinblastine. According to the current results, C3435T variant was not associated with HL outcome in two groups of patients one with complete remission and the other with relapse. However, previous reports have shown that the C3435T polymorphism alters the response in different cancers. For example, the wild type genotype CC was associated with better chemotherapy response in patients with NSCLC [32,33] and in patients with SCLC [34]. On the other hand, CC genotype was linked significantly with increased risk of relapse in AML patients [35]. Furthermore, our study revealed no significant association between progression free survival and C3435T genotype and allele frequencies. However, previous studies have shown the effect of C3435T variant on survival time in cancer patients. The CC genotype was associated with a shorter overall survival in patient's with multiple myloma [36] and in patients with ALL [22] compared to both CT and TT genotypes. This difference in the results may be related to the variation in the genetic background of the studied groups, or life style or due to other unknown factors.

Results of this study show no significant association between HL response and patient's characteristics such as age, gender, HL stage, specimen histology and presence or absence of B-symptoms. In addition, the distribution of C3435T genotypes and alleles was not associated with patient's characteristics. Therefore, possibilities exist that other polymorphisms in the MDR1 gene might be involved in modulating HL response to drugs in the Jordanian population. Thus, scanning the MDR1 gene to search for common and new variants in the Jordanian population is important for future pharmacogenetic studies in this population.

In conclusion, results of this study show that C3435T polymorphism is associated with susceptibility to HL in Jordanian population. However, this variant is not correlated with the drug response or clinical parameters in HL patients.

## Competing interests

The authors declare that they have no competing interests.

## Authors' contributions

NM, OK, OA, and AA carried out the molecular genetic studies, participated in the sequence alignment and drafted the manuscript. IOM participated in the sequence alignment. NM, OK, KA and OA participated in the design of the study and performed the statistical analysis. WH and IIM have participated in the study design and samples collection and preparation for perform the study. NM and KA helped to draft the manuscript. All authors read and approved the final manuscript.
